# Rationale and Design of the PASSIVATE‐CAP Trial: The Preventive Intervention Value of Drug‐Coated Balloons in Vulnerable Coronary Atherosclerotic Plaques

**DOI:** 10.1002/clc.70243

**Published:** 2026-01-11

**Authors:** Zhongxiu Chen, Junyan Zhang, Minggang Zhou, Shichu Liang, Yong Chen, Chen Li, Hua Wang, Jiafu Wei, Baotao Huang, Mian Wang, Yong He, for the PASSIVATE‐CAP trial investigator

**Affiliations:** ^1^ Department of Cardiology West China Hospital of Sichuan University Chengdu China

**Keywords:** acute coronary syndrome, drug‐coated balloon, percutaneous coronary intervention, vulnerable plaque

## Abstract

**Background:**

Patients with acute coronary syndrome (ACS) face a significantly increased risk of cardiovascular events due to vulnerable plaques. However, no clear evidence supports performing preventive percutaneous coronary intervention (PCI) for non‐flow‐limiting vulnerable plaques. To address this gap, the PASSIVATE‐CAP study was designed to investigate the therapeutic potential of drug‐coated balloon (DCB) for treating non‐flow‐limiting vulnerable plaques.

**Methods:**

The PASSIVATE‐CAP study is an investigator‐initiated, prospective, randomized, multicenter, open‐label superiority trial focusing on acute coronary syndrome (ACS) patients with non‐flow‐limiting vulnerable plaques in non‐culprit vessels. In this study, eligible patients will be randomized at a 1:1 ratio into two groups: patients who received guideline‐directed medical therapy (GDMT) and patients who received GDMT combined with a drug‐coated balloon (DCB). The primary endpoint was the minimal lumen area of the target lesion 1 year after randomization. The secondary endpoints encompass a range of factors, including the proportion of patients with vulnerable plaques in the target vessel, fibrous cap thickness, lipid core arc of the target lesion, minimal lumen area of the target vessel, among others. The study has been registered on Clinicaltrials.gov (Identifier: NCT06416813).

**Results:** The PASSIVATE‐CAP study enrolled its first patient on July 14, 2025, with projected enrollment completion by January 31, 2027. As of December 28, 2025, 6 patients have been enrolled. The anticipated study end date, marking the completion of the follow‐up period, is January 31, 2028.

**Conclusions:**

The PASSIVATE‐CAP study represents the first prospective, randomized, multicenter, open‐label trial designed to explore the therapeutic value of DCB for the treatment of vulnerable plaques within the ACS patient population.

## Graphical abstract

PASSIVATE‐CAP Research Overview and Flowchart.

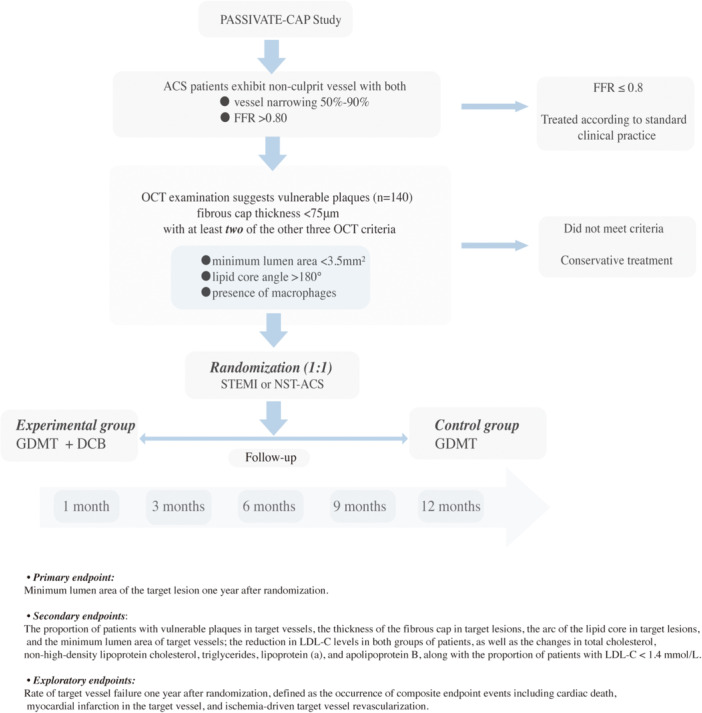

**Abbreviations**
ACS: acute coronary syndrome
ACT: activated clotting time
ASCVD: atherosclerotic cardiovascular disease
CRF: case record forms
DCB: drug‐coated balloon
EDC: electronic data capture
FFR: fractional flow reserve
GDMT: guideline‐directed medical therapy
MACE: main adverse cardiac events
PCI: percutaneous coronary intervention
PCSK‐9: proprotein convertase subtilisin/kexin type 9
QCA: quantitative coronary angiography
TCFA: thin‐cap fibroatheromas
TIMI: thrombolysis in myocardial infarction

## 1 Introduction

Acute coronary syndrome (ACS) is a leading cause of mortality among patients with cardiovascular diseases. Research indicates that patients with ACS consistently face the threat of non‐culprit lesions. On average, individuals with ACS have five non‐culprit lesions, and approximately 79% of these patients experience the rupture of one or more vulnerable plaques in non‐culprit vessels [1, 2].

Vulnerable plaques, atherosclerotic lesions prone to rupture, thrombus formation, and rapid progression to “culprit plaques” form the primary pathological basis for acute coronary syndrome (ACS). Research has shown that vessels with a negative fractional flow reserve (FFR) often contain vulnerable plaques, increasing the risk of clinical adverse events [3]. The COMBINE OCT‐FFR study demonstrated an elevated risk of major adverse cardiac events (MACEs) in patients with negative FFR but optical coherence tomography (OCT)‐identified vulnerable plaques during follow‐up [4]. Additionally, vulnerable plaques are associated with a 17%–19% incidence rate of MACEs, highlighting the critical need for targeted interventions aimed at these plaques to mitigate the risk of cardiovascular events [5, 6].

However, routine prophylactic stent implantation for non‐flow‐limiting vulnerable plaques is not recommended due to the lack of robust clinical evidence and the associated risks, including perioperative myocardial infarction, stent thrombosis, and in‐stent restenosis. Drug‐coated balloons (DCBs) have received a Class I recommendation for treating in‐stent restenosis [7]. Furthermore, a limited number of single‐center retrospective studies have gradually begun to indicate that DCBs may be as effective as drug‐eluting stents for treating culprit lesions in patients with ACS [8, 9]. At the same time, given the significant “leave nothing behind” advantage of DCBs, their clinical applications are continuously being promoted [10]. However, the preventive value of DCBs for vulnerable plaques has yet to be confirmed.

The objective of the present study, “Preventive intervention with drug‐coated balloon for vulnerable Coronary Atherosclerotic Plaques (PASSIVATE‐CAP)”, was to investigate the potential of DCB for preventing vulnerable coronary atherosclerotic plaques in non‐flow‐restricted non‐culprit vessels in ACS patients. By proposing a novel strategy accompanied by practical guidance, this study aimed to address the preventive treatment needs of a high‐risk population susceptible to recurrent ACS events.

## 2 Methods

### 2.1 Study Design

**Figure 1:**
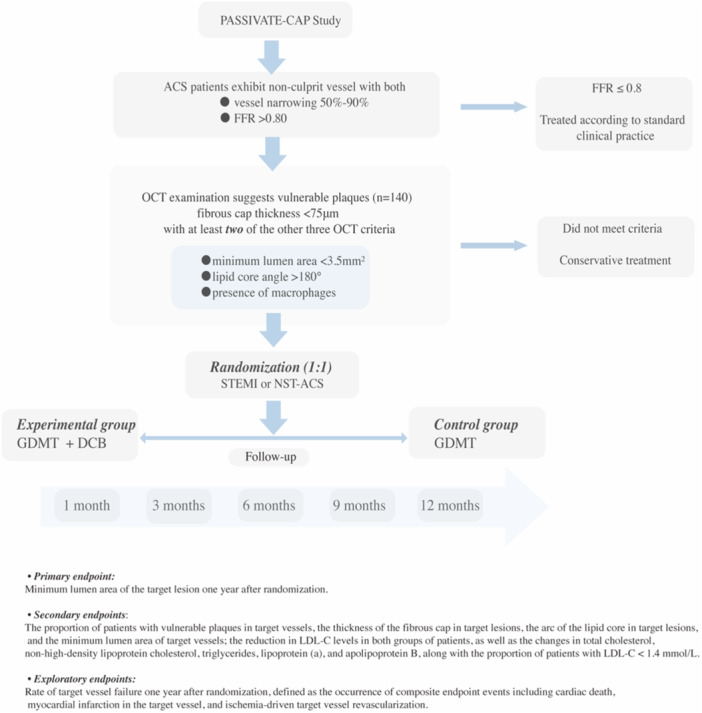
Flow chart of the study.

The PASSIVATE‐CAP trial, a prospective, multicenter, randomized, open‐label, superiority trial, aims to evaluate the therapeutic value of DCB for treating vulnerable plaques. This trial was approved by the West China Hospital of Sichuan University in Chengdu, China. A detailed flowchart of the trial design is illustrated in Figure 1. The study protocol received approval from the institutional review boards at all participating centers and was conducted in strict accordance with the Declaration of Helsinki [11]. Additionally, the study has been registered on Clinicaltrials. gov (Identifier: NCT06416813). The trial aims to enroll approximately 140 patients across five subcenters in China (see Supplementary Appendix). To qualify, each participated medical institution must meet specific criteria, including an annual percutaneous coronary intervention (PCI) volume of at least 500 cases. Additionally, the primary operator must hold valid interventional treatment qualifications and have experience independently performing at least 100 PCIs annually, ensuring proficiency in conducting emergency PCIs. Operators must also proficiently master the procedural execution and interpretation of the results obtained via OCT and FFR. The trial had launched in June 26, 2025, and will be anticipated a case inclusion period of 12–18 months. Subcenters will compete for enrollment and each subcenter will be capped at a maximum enrollment of 70 patients.

### 2.2 Study Population

The specific inclusion and exclusion criteria for the study are detailed in Table 1. This study will target patients with ACS who have vulnerable plaques in non‐culprit vessels and do not exhibit flow restriction. Participants meeting the eligibility criteria will be allocated in one of two groups: the guideline‐directed medical therapy (GDMT) group, which will adhere to standard lipid‐lowering strategies as recommended by current guidelines; or the GDMT combined with DCB treatment group [12]. The latter group will follow the same lipid‐lowering strategy as the GDMT group but will additionally receive DCB as a preventive intervention for the target lesion. Informed consent will be secured from all participants, with follow‐up assessments scheduled at predetermined intervals (detailed patient informed consent principles see Supplementary Appendix).

**Table 1:** Inclusion and exclusion criteria for this study.

Criteria	Inclusion	Exclusion
**Basic Information**	Age ≥ 18 years; Patients diagnosed with ACS (STEMI, NSTEMI, and high‐risk unstable angina^a^) who underwent PCI for the culprit vessel; Patients provide informed consent and are willing to complete follow‐up and examinations as per study protocol; Life expectancy > 1 year.	Patients with cardiogenic shock, hemodynamic or electrical instability; Patients with contraindications for FFR assessment medications (e.g., asthma); Advanced heart failure (NYHA Class III‐IV); Ischemic stroke in the past 6 months or any history of intracranial hemorrhage; Severe valvular disease or valvular disease requiring surgical intervention or percutaneous valvular replacement; Previous target vessel myocardial infarction or PCI target coronary artery revascularization, or history of CABG; Planned major surgery requiring interruption of dual antiplatelet therapy; Severe liver dysfunction (Child‐Pugh Class C), eGFR < 30 mL/min/1.73㎡; Comorbidities that may affect the completion of the study process; Other conditions that deem the patient unsuitable for inclusion in clinical trials (e.g., pregnancy or lactation, participation in another clinical trial that interferes with the primary endpoints of this study, poor compliance) etc.
**Angiographic and OCT Results**	Single diameter stenosis of 50%‐90% in the major coronary artery segment (diameter 2.75–4 mm) of non‐culprit lesions (target vessel segment must be in situ without prior PCI treatment, not a venous or arterial graft); FFR value greater than 0.8; OCT findings indicating lipid‐rich plaque, fibrous cap thickness < 75 µm, plus at least 2 of the other 3 OCT criteria (minimal lumen area < 3.5 mm², lipid arc > 180°, presence of macrophages); Lesion length ≤ 30 mm. Target lesions must be located in different coronary branches (two target lesions in one vessel that can be covered by one drug‐coated balloon are eligible).	TIMI flow < 2 in the culprit vessel post‐PCI; Patients with more than three target lesions; left main disease; ostial lesions; thrombotic lesions; severely calcified lesions (where devices cannot pass or require rotational atherectomy); true bifurcation lesions requiring stent implantation; Coronary anatomy (severe calcification^b^, tortuosity, etc.) preventing complete imaging of the segment of interest (including at least 5 mm from both edges of the stenosis), coronary anatomy not suitable for PCI or OCT assessment; Coronary artery diffuse disease or presence of ≥ 1 untreated non‐culprit lesion (non‐culprit flow‐limiting lesions planned for subsequent staged PCI, but processed patients can be included).

Abbreviations: ACS, acute coronary syndrome; CABG, coronary artery bypass grafting; DAPT, dual antiplatelet therapy; eGFR, estimated glomerular filtration rate; FFR, fractional flow reserve; NSTEMI, actue non‐ST segment elevation myocardial infarction; NYHA, New York Heart Association; OCT, optical coherence tomography; PCI, percutaneous coronary intervention; STEMI, acute ST‐Segment elevation myocardial infarction; TIMI, thrombolysis in myocardial infarction.

^a^ High‐risk unstable angina was defined as worsening anginal symptoms accompanied by ischemic ST‐T changes or elevation of cardiac biomarkers not exceeding twice the upper limit of normal.

^b^ Severe calcification in angiography is defined as visible calcification on both sides of the vessel wall in the absence of cardiac motion.

### 2.3 Randomization

Eligible patients who met the inclusion and exclusion criteria will be randomly assigned to either the ‘GDMT group’ or the ‘GDMT combined with DCB group’ at a 1:1 ratio. This randomization process will be stratified based on the presence or absence of ST‐Segment Elevation Myocardial Infarction (STEMI) to ensure balanced allocation. The randomization procedure will take place in the interventional catheterization room at each study center and will be designed to be completed within a 2 min timeframe. Treating physicians will utilize an interactive web‐based response system (IWRS) managed by a third party to facilitate random allocation and track the progress of the enrolled participants.

### 2.4 Standard Treatment

All patients enrolled in the study will be adhered to routine drug treatment protocols as outlined in current guidelines [13]. For those not already receiving antiplatelet therapy, a loading dose of aspirin and a P2Y_12_ inhibitor will be administered. Prior to PCI, patients will receive an intravenous dose of unfractionated heparin at 100 U/kg. During the procedure, additional doses of unfractionated heparin may will be administered as necessary, with the goal of maintaining the activated clotting time (ACT) within the range of 200–250 s, taking into account factors such as procedural duration and lesion complexity. The pre‐PCI use of glycoprotein IIb/IIIa receptor antagonists will be prohibited, and their routine application during and after PCI is not advised, except for rescue purposes at the interventionalist's discretion, based on lesion characteristics. Routine post‐PCI anticoagulation is generally not recommended unless the PCI physician identifying a specific need. Following PCI, all participants continued on standard doses of aspirin and P2Y_12_ inhibitors (clopidogrel or ticagrelor) for antiplatelet therapy. Guideline‐recommended medications, including beta‐blockers, and angiotensin‐converting enzyme inhibitors/angiotensin II receptor blockers will be tailored and prescribed according to each patient's condition in both groups [14]. For patients already on oral anticoagulants or meanwhile needed anticoagulant therapy (such as DOACs or VKAs), a short‐term triple antithrombotic therapy regimen, varying from 1 week to 1 month determined by the clinician's assessment for the risk of recurrent myocardial ischemic events, will be recommended according to the 2021 North American Perspective [15] and our routine clinical practice. This is followed by a combination of clopidogrel and anticoagulation for approximately 6 months to 1 year (determined by the clinician's balancing the ischemia and bleeding risk), after which lifelong anticoagulation therapy will be continued. All subjects are scheduled for regular follow‐ups to monitor their condition and adjust their medication use as necessary.

### 2.5 Interventional Procedure Process

Initially, the interventionist addresses the culprit vessels in patients with ACS, and the treatment strategy will be determined at the discretion of the operator based on current guidelines and the condition of the lesions [13]. The use of intracoronary imaging techniques, such as OCT, will be recommended for lesion assessment and procedural optimization when necessary; however, the final decision on whether to employ these techniques rests with the operator. Following the management of the culprit lesions, non‐culprit vessels exhibiting stenosis with 50% to 90%, as indicated by coronary angiography, will undergo fractional flow reserve (FFR, Abbott) analysis. If the FFR value exceeds 0.8, OCT (Abbott) will be utilized to evaluate plaque vulnerability. Patients with vulnerable plaques who meet the inclusion criteria and do not meet any exclusion criteria will be considered for trial enrollment. Functional and morphological evaluation of non‐culprit vessels will be performed either during primary PCI or as a staged procedure before discharge, depending on the patient's clinical condition. Pre‐discharge assessment is encouraged; if deferred post‐discharge, it should be completed within 1 month. For STEMI patients, FFR measurement will be performed ≥6 days after initial presentation.

### 2.6 Lipid‐Lowering Strategy in the GDMT Group

In this study, the ASCVD risk stratification was conducted in accordance with the 2023 China Guidelines for Lipid Management, which categorizes ASCVD risk into five levels: low, moderate, high, very high, and extremely high [16]. Based on the presence of ASCVD, prevention is categorized into primary and secondary, with the former encompassing low, moderate, and high risks, and the latter comprising very high and extremely high risks. All patients included in this study are ACS patients, and therefore, will be categorized as either very high or extremely high risk. Among them, patients who have experienced ≥ 2 severe ASCVD events or have had 1 severe ASCVD event and ≥ 2 high‐risk factors will be classified as extremely high risk, with the rest being very high risk (Definitions of severe ASCVD events and high‐risk factors are provided in Table 2). For patients with very high‐risk ASCVD, the target LDL‐C level is < 1.8 mmol/L with a ≥ 50% reduction from baseline, and for those with additional high‐risk factors, classified as extremely high risk ASCVD patients, the target LDL‐C level is < 1.4 mmol/L with a ≥ 50% reduction [16].

**Table 2:** Serious ASCVD events and high‐risk factors.

Serious ASCVD event and High‐risk factors
Serious ASCVD event
Recent history of Acute Coronary Syndrome (ACS) (< 1 year)
History of prior myocardial infarction (apart from the abovementioned ACS)
History of ischemic stroke
Symptomatic peripheral vascular disease, having undergone prior vascular reconstruction or amputation
High‐risk factors
LDL‐C ≤ 1.8 mmol/L with recurrence of serious ASCVD events
Early onset coronary heart disease (men < 55 years, women < 65 years)
Familial hypercholesterolemia or baseline LDL‐C > 4.9 mmol/L
History of CABG (Coronary Artery Bypass Grafting) or PCI (Percutaneous Coronary Intervention) treatment
Diabetes
Hypertension
Chronic Kidney Disease stages 3 to 4
Smoking

Specific treatment recommendations will be stratified by LDL‐C levels and statin use history, including lifestyle adjustments, statin therapy initiation or intensification, and the addition of lipid‐lowering agents such as ezetimibe or PCSK9 inhibitors, including PCSK9 monoclonal antibodies or Inclisiran, as necessary (Supplementary Appendix). According to the 2023 China Guidelines for Lipid Management, PCSK‐9 inhibitors are primarily indicated for patients whose LDL‐C levels remain above target despite the use of moderate‐intensity statins in combination with a cholesterol absorption inhibitor (Class I recommendation). Additionally, other scenarios may warrant consideration for initiating PCSK‐9 inhibitor therapy, such as extremely high‐risk patients with elevated baseline LDL‐C levels who are unlikely to achieve target levels with statin therapy combined with a cholesterol absorption inhibitor, as well as patients who are intolerant to statins (Class IIa recommendation) [16].

### 2.7 The GDMT Combined With DCB Group

The lipid‐lowering strategy for patients in the GDMT combined with DCB group mirrors that of patients in the GDMT group. For the treatment of vulnerable plaque lesions using a DCB, the protocol was as follows: The target lesion was first predilated using a semi‐compliant conventional balloon, aiming for a balloon‐to‐vessel diameter ratio of 1:1. This may be supplemented with special balloons (such as noncompliant, cutting, or scoring balloons) as necessary. Following pre‐dilation, the degree of residual stenosis in the target vessel was evaluated. Patients who exhibit successful pretreatment outcomes (defined as residual stenosis ≤ 30% without flow‐limiting dissection) will proceed to DCB treatment using a paclitaxel‐coated balloon (Sequent® Please, B. Braun, Germany, with a paclitaxel drug content of 3 μg/mm^2^ on the DCB surface). The diameter and length of the DCB are selected based on measurements of the distal diameter and length of the target vessel lesion. The DCB should extend 2‐3 mm beyond the pre‐dilation area at both ends, maintaining a balloon/vessel diameter ratio of 1:1, with a minimum dilation time of 60 s. If a flow‐limiting dissection (> C grade) occurs, rescue stent implantation in the target vessel is permitted. OCT evaluation prior to DCB deployment was encouraged but non‐mandatory. The frequency of rescue stent implantation was factored into the statistical analysis.

### 2.8 Primary Outcome

The primary endpoint of this study is defined as the minimum luminal area of the target lesion measured 1 year after randomization.

### 2.9 Secondary Outcome

The secondary endpoints of this study encompass a range of factors. These included the proportion of patients with vulnerable plaques in the target vessel, the fibrous cap thickness and lipid core arc of the target lesion plaque, and the minimum luminal area of the target vessel during follow‐up. The study will also assess changes in total cholesterol, non‐high‐density lipoprotein cholesterol, triglycerides, lipoprotein (a), and apolipoprotein B levels, as well as the proportion of patients achieving an LDL‐C level less than 1.4 mmol/L.

### 2.10 Exploratory Outcomes

An exploratory research endpoint of this study is to determine the incidence rate of target vessel failure within 1 year following randomization. Target vessel failure will be conceptualized as a composite endpoint event encompassing cardiac death, target vessel myocardial infarction, and ischemia‐driven target vessel revascularization. For the specific definitions of exploratory outcomes, see the Supplementary Appendix.

### 2.11 Safety Assessment

There is a potential risk of vascular acute occlusion or thrombosis due to the absence of stent strut support following DCB treatment. The procedural maneuvers of DCB should adhere to the recommendations from an Asia‐Pacific Consensus Group [17] and the Third Report of the International DCB Consensus Group [18]. A satisfactory lesion preparation will be attributed to the improved vascular positive remodeling. Extend the observation period (10 min) after DCB balloon withdrawal should be conducted and followed by an angiogram to ensure satisfactory result. In cases where subjects experience Thrombolysis In Myocardial Infarction flow < 3, severe dissection (type D, E, and F), a rescue drug‐eluting stent will be implanted mandatorily for bailout treatment. More details see Supplementary Appendix.

### 2.12 Follow‐Up

All patients will be scheduled to undergo four on‐site follow‐up visits at 1, 3, 9 months, and 1‐year post‐inclusion. Additionally, they received a telephone follow‐up at 6 months. At the 1‐year mark, all participants will be required to be admitted to the hospital for repeat coronary angiography and OCT of the targeted vessels. Core lab (Core Laboratory of Cardiovascular Interventional Imaging at the National Center for Cardiovascular Diseases), blinded to the study design, will analyze and adjudicate the OCT parameters. Details of the follow‐up visits see Supplement Appendix.

### 2.13 Sample Size Estimation

The PROSPECT ABSORB study [19] aimed to seal and passivate vulnerable plaques using bioresorbable scaffolds. Research has revealed that at a follow‐up of 25 months, compared to GDMT alone, the combination of GDMT and bioresorbable scaffold treatment results in a significantly larger minimum lumen area (6.9 ± 2.6 mm^2^ vs. 3.0 ± 1.0 mm^2^). The sample size estimation for this study is based on the primary effectiveness endpoint. According to the PROSPECT ABSORB study, at the 1‐year follow‐up, the minimum lumen area of the target lesion in the “GDMT group” was conservatively assumed to be 3.0 ± 2.6 mm^2^, while the conservative estimate for the “GDMT combined with DCB group” was 4.5 ± 2.6 mm^2^. Using Two‐Sample Z‐Tests Allowing Unequal Variance in PASS 2023, with a difference of 1.5, an α set at 0.05 (two‐sided), and a test power of 90%, the required sample size per group was 63 patients, with a total sample size of 126 patients. Assuming a dropout rate of 10%, the target sample size is approximately 140 patients, with 70 patients in each group.

### 2.14 Statistical Analysis

Blinded and independent statistics team will perform the statistical analysis. Comparisons between the two treatment groups in this study will be adhere to the intention‐to‐treat principle. Careful trial planning conducting will minimize the occurrence of missing data as far as possible. The details for handling of missing values see Supplementary Appendix. For categorical variables, the data are presented as numbers and percentages, with comparisons conducted using either the chi‐square test or Fisher's exact test, depending on the data distribution. Continuous variables will be reported as either the mean ± standard deviation or median with interquartile range. The comparison of the primary endpoint between the treatment groups will be performed using a t‐test. For secondary endpoints, the T test or chi‐square test will be applied as appropriate. The cumulative event rates for exploratory endpoints will also be compared using the chi‐square test or Fisher's exact test. The time‐to‐event analysis will be performed using the Kaplan–Meier method, with differences in event‐free survival between Kaplan–Meier curves compared using the log‐rank test. Subgroup analyses for the primary endpoint will consider factors such as ACS subtypes, diabetes status, PCSK9 inhibitor usage, lesion length greater than 10 mm, and vessel diameter greater than 3 mm, among other relevant factors. There are no substudies planned for this trial.

### 2.15 Study Governance and Oversight Committees

The PASSIVATE‐CAP trial operates under a structured governance framework to ensure scientific integrity and participant safety.

The Executive/Steering Committee, co‐chaired by Yong He and Zhongxiu Chen, comprises principal investigators from all participating centers (Supplementary Appendix). This committee oversees trial conduct, monitors study progress, and makes key operational decisions throughout the trial duration.

An independent Data Safety Monitoring Board (DSMB) of three members (Supplementary Appendix) reviews accumulating safety data and interim results at predefined intervals. Operating independently from investigators and sponsors, the DSMB provides recommendations to the Executive Committee regarding trial continuation, modification, or early termination as warranted.

The Endpoint Adjudication Committee independently adjudicates all primary and secondary clinical endpoints‐including cardiovascular death, myocardial infarction, and target vessel revascularization‐using predefined criteria while blinded to treatment allocation, ensuring unbiased outcome assessment.

## 3 Discussion

PASSIVTAE‐CAP is a prospective, multicenter, randomized, open‐label, superiority trial to evaluate the therapeutic value of DCB for treating vulnerable plaques. The recurrence of cardiovascular events in ACS patients is intricately linked to LDL‐C levels and the progression of plaque, with vulnerable plaques significantly elevating the risk of such events [19, 20]. Given the prevailing view that intensified lipid‐lowering strategies play a vital role in managing such patients, questions persist regarding the necessity of further reducing lipid levels beyond current recommendations and the potential benefits of preventive interventions.

This trial focuses on a population of ACS patients and used FFR and OCT as tools for assessing coronary physiology and plaque characteristics; vulnerable plaques in non‐flow‐limiting noncriminal vessels will be the intervention targets. New strategies with practical value for preventing recurrent ACS in high‐risk populations are proposed.

**1. The Potential Hazards of Non‐Culprit Lesions in ACS Should Not Be Overlooked**

ACS is a significant and prevalent cause of cardiovascular mortality. Research indicates that patients with ACS face a continuous threat from non‐culprit lesions. A pivotal three‐vessel intravascular ultrasound study by Rioufol et al. revealed that, on average, each ACS patient harbors five non‐culprit lesions, 79% of which rupture one or more vulnerable plaques in non‐culprit vessels [1, 2]. These findings highlight the persistent nature of non‐culprit lesions and their association with an elevated risk of cardiovascular events. An observational study involving 697 ACS patients who underwent PCI showed that the incidence of MACEs within 3 years post‐PCI was 20%, with the majority (over 60%) of these events occurring within the first year. Moreover, a study revealed that the recurrence risk of events related to non‐culprit lesions was 11.6%, closely mirroring the 12.9% risk associated with culprit lesion‐related events [21]. These data emphasize the significant contribution of non‐culprit lesions to the overall incidence of MACEs in ACS patients, highlighting the critical need to address the potential hazards of these lesions.

**2. Vulnerable Plaques Significantly Increase the Risk of Cardiovascular Events**

Vulnerable plaques, which are prone to rupture and thrombus formation and can rapidly evolve into “culprit plaques,” form the primary pathological basis of ACS. Clinically, intravascular imaging revealed that some patients may only exhibit mild lesions on coronary angiography but are found to have thin‐cap fibroatheroma (TCFA). This finding suggested that even non‐flow‐limiting coronary vulnerable plaques carry a risk for adverse events [3]. In the COMBINE OCT‐FFR study, ACS patients with diabetes initially received interventions for culprit lesions. Subsequently, all non‐culprit lesions showing 40%–80% stenosis on coronary angiography underwent FFR assessment. Lesions with a positive FFR (FFR ≤ 0.80) will revascularized, whereas those with a negative FFR (FFR > 0.80) were further evaluated using OCT. This study demonstrated that FFR‐negative lesions with TCFA, as detected by OCT, were associated with a significant increase in MACE during follow‐up [4]. Similarly, the CLIMA study revealed that patients with OCT‐defined high‐risk intermediate lesions (minimum lumen area < 3.5 mm^2^, fibrous cap thickness < 75 µm, lipid arc circumferential extension > 180°, and OCT‐defined macrophages) had a substantially greater rate of cardiac death or myocardial infarction in the target left anterior descending segment at 1 year than patients without such vulnerable plaques (19.4% vs 3.1%, *p* < 0.001; HR = 7.54 (3.1–18.6)) [6]. Moreover, Jiang et al., through OCT examinations of three vessels in 883 patients undergoing primary PCI, corroborated these findings, noting an incidence rate of adverse events as high as 17.4% in the vulnerable plaque group [5]. Thus, stabilizing or reducing vulnerable plaques could theoretically decrease the likelihood of adverse cardiovascular events.

**3. Evaluation of Non‐Culprit Vulnerable Lesions and Revascularization Strategies in Acute Coronary Syndrome Patients**

The PROSPECT ABSORB study utilized bioresorbable scaffolds to treat lesions characterized by non‐flow limitation but with an intravascular ultrasound indication of plaque burden greater than 65%. This study focused primarily on the minimal lumen area in the target vessel at the 25‐month follow‐up [19]. The findings revealed that, compared to optimized medical therapy, bioresorbable scaffold treatment resulted in a greater minimal lumen area without increasing the rate of target lesion failure and showed a trend toward reducing the risk of MACE associated with the target lesion. The PREVENT study revealed that, compared with optimal medical therapy alone, sealing and passivating vulnerable plaques by bioresorbable scaffolds or polymer‐coated everolimus‐eluting stents reduced MACE arising from high‐risk vulnerable plaques. However, the therapeutic value of preventive PCI in concert with more potent pharmacotherapies, such as PCSK9 inhibitors, needed further evaluation. Moreover, while interventions on vulnerable plaques contribute to their passivation and stabilization, routine prophylactic interventions are not currently recommended due to the lack of robust clinical evidence and the associated risks of perioperative myocardial infarction, in‐stent thrombosis, and restenosis associated with stent implantation. Zimmermann et al. noted that the recommendation for prophylactic intervention in non‐flow‐limiting plaques requires a careful balance between the risk of the plaque and the risk of PCI [22]. Preventive PCI for non‐flow‐limiting plaques is considered only if the risk of PCI is significantly lower than the risk of adverse events due to plaque progression [22]. The use of DCBs may have this potential. DCBs coat the balloon surface with a lipid‐soluble, rapidly absorbable drug that persistently inhibits cell growth, enabling uniform transfer of the drug to the vessel wall during balloon expansion [7]. Given their larger surface area, more uniform drug transfer compared to the limited stent strut area and uneven drug delivery. Animal study showed that DCB treatment of non‐obstructive plaques led to a reduction of inflammation and plaque burden [23]. Moreover, DCBs avoid the permanent presence of metallic stent struts and potential inflammatory reactions induced by polymer coatings and are recommended as a Class I indication for treating in‐stent restenosis [7]. The unique appeal of DCBs for ‘no foreign body implantation’ and their positive vessel remodeling advantages have also led to their use in treating small de novo coronary lesions. However, their prophylactic value in treating vulnerable plaques has yet to be confirmed.

**4. The Value of Intensified Lipid‐Lowering Strategies in Treating Vulnerable Plaques**

Currently, there are no universally accepted guidelines recommending treatment strategies specifically for vulnerable plaques. Studies of EASY‐FIT [24], YELLOW [25], HUYGENS [26], and PACMAN‐AMI [27] have revealed that intensified lipid‐lowering is beneficial for stabilizing and reversing vulnerable plaques. However, existing guidelines universally adopt a lipid‐lowering strategy based on risk stratification and target lipid levels in atherosclerotic patients without targeting vulnerable plaques with the potent use of PCSK9 inhibitors [12, 13].

**5. Potential Mechanisms of DCBs in the Treatment of Vulnerable Plaques**

Based on the current body of research, it can be proposed that DCBs may exert therapeutic effects on vulnerable plaques through two primary mechanisms [28]. Firstly, the mechanical expansion of the balloon during angioplasty may facilitate the redistribution of plaque material, which could contribute to lesion management. This process has the potential to reduce plaque volume and improve local blood flow, potentially enhancing the stability of the vascular wall and decreasing the risk of adverse coronary events. Secondly, the pharmacological agents released from DCBs may offer various beneficial properties, including anti‐inflammatory and antiproliferative effects on smooth muscle cells [28]. These effects may directly address some of the underlying pathological mechanisms associated with vulnerable plaques by inhibiting abnormal smooth muscle cell proliferation and attenuating local inflammatory responses, thereby promoting a more stable plaque environment [28]. Furthermore, the sustained drug release characteristics of DCBs might provide advantages in targeted therapy, potentially yielding prolonged therapeutic effects over time. The ongoing DEBuT‐LRP Trial (NCT04765956), which examines the safety and effectiveness of DCB for the treatment of lipid‐rich plaques, could provide important insights for future treatment strategies.

In conclusion, vulnerable plaques are a significant risk factor for cardiovascular events, underscoring the need for effective management strategies in patients with ACS. Recent advancements have introduced PCSK9 inhibitors, which offer new avenues for enhancing lipid management and reducing cardiovascular risk. Concurrently, the non‐implantation approach and the positive vascular remodeling offered by DCBs present promising alternatives to traditional treatments. Against this backdrop, the PASSIVATE‐CAP trial seeks to explore the preventive interventional potential of DCB in concert with contemporary intensified lipid‐lowering strategies including more potent pharmacotherapies (PCSK9 inhibitors) for treating vulnerable plaques in non‐flow‐limiting, non‐culprit vessels of ACS patients. This approach aims to establish new, practically applicable strategies for the preventive treatment of patients at high risk of recurrence myocardial infarction, leveraging the unique advantages of both pharmacological and interventional therapies to address the residual lesion of ACS.

## 4 Limitation

This study has several limitations that warrant consideration. First, as an open‐label trial, the lack of blinding for treatment allocation may introduce performance and detection bias, although the use of blinded core laboratory analysis for OCT endpoints and an independent adjudication committee partially mitigates this concern. Second, the relatively small sample size and single‐country recruitment may limit the generalizability of findings to broader populations with different genetic backgrounds and clinical practices. Third, the study population is restricted to ACS patients with specific OCT‐defined vulnerable plaque characteristics, which may not represent the full spectrum of high‐risk coronary lesions encountered in clinical practice.

## Conflicts of Interest

The authors declare no conflicts of interest.

## Supporting information

supplementary appendix _revised 6–30 docx.docx.

## Data Availability

The data underlying the findings of the paper are freely available on request through the authors themselves. Yong He (Department of Cardiology, West China Hospital of Sichuan University, 37 Guo Xue Xiang, Chengdu, Sichuan, 610041, China; e‐mail: heyong_huaxi@163. com) was contacted to request the data.
