# Supplementary material for: Rationale and Design of the PASSIVATE‐CAP Trial: The Preventive Intervention Value of Drug‐Coated Balloons in Vulnerable Coronary Atherosclerotic Plaques
Source: Clin Cardiol. 2026 Jan 11;49(1):e70243. doi: 10.1002/clc.70243 (PMC12791565; doi:10.1002/clc.70243)
Supplement: Supplementary file 1 — supplementary appendix _revised 6–30 docx.docx. [file CLC-49-e70243-s001.docx]

**Supplementary Appendix**

**Rationale and Design of the PASSIVATE-CAP trial: the Preventive Intervention Value of Drug-Coated Balloons in Vulnerable Coronary Atherosclerotic Plaques**

**Hypothesis and objectives**

The primary hypothesis is that, compared with the optimized lipid-lowering therapy recommended by existing guidelines, employing DCB treatment based on an optimal medication strategy in patients with ACS is expected to increase target luminal patency at 1 year post randomization. Secondary hypotheses are that the “GDMT combined DCB” therapy in ACS patients will achieve lower lipid level (such as LDL-C), higher proportion of patients achieving prespecified LDL-C goals (LDL-C < 1.4 mmol/L), stabilization vulnerable plaques, and reducing the proportion of vulnerable plaques in target vessels in comparison to the GDMT approach at 1 year. The primary objective is to determine whether the “GDMT combined DCB” therapy is superior to GDMT approach in terms of primary endpoints in patients with ACS. The secondary objective is to determine whether the “GDMT combined DCB” therapy is superior to GDMT approach in terms of secondary endpoints in patients with ACS.

**Research Innovations**

The innovation of this study lies primarily in the novelty of its therapeutic concepts and application strategies, encompassing three key areas:

**Optimization of Early and Intensive Lipid-Lowering Treatment Strategies Based on Current Treatment Protocols**

While guidelines and expert consensuses advocate for intensified lipid-lowering therapy in patients with ACS, the method for accessing lipid targets merits further exploration. Given the 4- to 6-week intervals for LDL-C evaluation and treatment escalation, it may take up to three months for patients to reach LDL-C targets, a period that coincides with the highest risk period for recurrent cardiovascular events. The "Strike early and strike strong" lipid-lowering philosophy, suggested by the 2022 ACVC, EAPC, and ESC Clinical Expert Consensus for early lipid management post-ACS, considers combining PCSK9 inhibitor treatment for very high-risk ASCVD patients during the acute phase. Although this concept is rational, further support from clinical evidence-based medicine is needed. Currently, there are no recommendations for intensified lipid-lowering strategies targeting vulnerable plaques. This project innovatively explored the value of combining intensified lipid-lowering (including use of PCSK9 inhibitors) and DCB for sealing and attenuating vulnerable plaques. The results could provide new directions and evidence-based medicine for the treatment of ACS caused by non-culprit vessel vulnerable plaques.

**Optimization of Interventional Treatment Plans for Patients in the High-Risk Group of Recurrent Cardiovascular Events in ACS Patients**

There is a lack of research on preventive interventional treatment with DCB for ACS patients with non-culprit vessel vulnerable plaques. The potential value of DCB in concert with intensive lipid-lowering treatment in treating non-culprit vessel vulnerable plaques in ACS patients are worthy research directions. This project innovatively targets non-culprit vessel vulnerable plaques in ACS patients using FFR and OCT for vascular physiological function and plaque morphology assessment to explore the attenuating value of DCB treatment. The results can be directly applied in the clinical management of ACS patients, representing a significant clinical application innovation.

**Exploration of Optimal Interventional Treatment Plans for Vulnerable Plaques by Combining Intravascular Imaging and Functional Tests**

This project used OCT to rapidly identify non-culprit vessel vulnerable plaques in ACS patients, providing DCB plaque-attenuating treatments for potentially unstable high-risk plaques, aiming to increase the patency of lumen and reduce adverse cardiovascular events such as recurrent myocardial infarction. By relying on OCT detection and integrating it with patients' clinical high-risk characteristics for comprehensive judgment, earlier and faster intervention strategies can be chosen. Moreover, based on an existing patient database, by analyzing clinical structured data and imaging data, a prognostic prediction model for patients could constructed, offering a more powerful technical tool for risk stratification and precise intervention.

**Consent**

Written informed consent to enter the trial and be randomized will be obtained from participants or, in the case of those lacking capacity to consent, from next of kin with legal responsibility. Consent will be obtained after the aims, methods, benefits, and potential risks of the trial have been explained, and prior to the performance of any trial-specific procedures or the collection of any blood samples for the trial. Once the patient regains the capacity to consent, persons enrolled via surrogate consent will be reconsented, with care taken to ensure that they understand that they are (1) free to withdraw from the research study and (2) withdrawn from the study will not jeopardize their future care. It will be made unambiguously clear that the participant (or guardian) is free to refuse to participate in all or any aspect of the research trial, at any time and for any reason, without incurring any penalty or affecting their access to standard treatment available at the recruiting site. The original signed consent forms will be signed by the investigator and documented in the electronic case report form (eCRF), a copy given to the participant or family and a copy placed in the participant’s medical notes.

**Study committees**

**Executive Committee**

The Executive Committee is composed of Yong He (Chairperson), Zhongxiu Chen, Duolao Wang (senior statistician, UK), Ranzun Zhao, Yong Zeng, Gang Li, Lin Cai, and Qiang Xue. This approach provides a scientific direction for the study and assesses the progress of the study. The Executive Committee will meet periodically and be assisted by the Steering Committee. The executive committee chairperson is responsible for communicating with the DSMB and sponsor when appropriate.

**Steering Committee**

The Steering Committee is composed of the executive committee and investigators from all participating centers, usually the principal investigators of every center. The Steering Committee will meet periodically to assess progress, provide scientific input and address policy issues and operational aspects of the protocol. Representatives of the sponsor may attend these meetings as nonvoting members.

**Data and Safety Monitoring Board**

An independent Data and Safety Monitoring Board (DSMB) will monitor the accruing safety and outcome data regularly. The DSMB will be composed of Qing Zhang (independent cardiologist), Ning Guo (independent interventionist), and Guanjian Liu (independent biostatistician). The DSMB operations will be formally separated from the sponsor, the investigators and the steering/executive committee. The DSMB will advise the chairperson of the executive committee by providing recommendations on trial continuation/discontinuation or aspects of study conduct.

**Lipid-lowering strategy in the GDMT group**

For ACS patients enrolled without additional high-risk factors and with LDL-C levels less than 1.8 mmol/L (70 mg/dl), those who have been regular statin users for more than four weeks are advised to intensify lifestyle interventions, such as dietary modifications, with adjustments to medication as necessary. In contrast, those who have been irregular statin users should initiate statin therapy. If LDL-C levels at enrollment are between 1.8 mmol/L (70 mg/dl) and 3.4 mmol/L (130 mg/dl), regular statin users should consider augmenting their treatment with additional lipid-lowering agents, such as ezetimibe or PCSK9 inhibitors. Irregular statin users, in this scenario, are recommended to start statin monotherapy. For patients presenting with LDL-C levels greater than 3.4 mmol/L (130 mg/dl), the recommendation is to either commence statin monotherapy or combine statin therapy with non-statin lipid-lowering drugs, including ezetimibe and/or PCSK9 inhibitors.

For ACS patients enrolled with additional high-risk factors and LDL-C levels less than 1.4 mmol/L (55 mg/dl), he recommendations for both regular and irregular statin users are aligned. When LDL-C levels at enrollment fall between 1.4 mmol/L (55 mg/dl) and 2.6 mmol/L (100 mg/dl), it is advised that regular statin users augment their regimen with additional lipid-lowering drugs, whereas irregular statin users should initiate statin monotherapy. For those presenting with LDL-C levels greater than 2.6 mmol/L (100 mg/dl), recommendations for initiating statin therapy or combining it with non-statin lipid-lowering agents are consistent across the board.

Participants in this study who were not regularly using statins prior to enrollment were initially advised to start with moderate-intensity statins. Should the lipid levels fail to reach the target after 4-6 weeks, the treatment strategy will be adjusted to include a combination of ezetimibe and/or PCSK9 inhibitors. For patients presenting high baseline LDL-C levels and anticipated challenges in achieving target levels with statins alone, combined treatment with statins and ezetimibe and/or PCSK-9 inhibitors can be initiated. Those intolerant to statins may opt for cholesterol absorption inhibitors or start directly with PCSK-9 inhibitors treatment.

**The specific definitions of exploratory outcomes**

*Death* was classified as cardiovascular death (including cardiac death and vascular death) or non-cardiovascular death. All causes of death were considered cardiac unless a clear noncardiac cause could be identified.

*Cardiac death:* Any death caused by cardiac reasons (such as myocardial infarction, low-output heart failure, fatal arrhythmias), unconscious death, death with an unknown cause, or all deaths related to PCI and associated treatments will be categorized as cardiac death.

*Vascular* death: Patients who died from noncoronary vascular conditions, such as cerebrovascular disease, pulmonary embolism, rupture of an abdominal aortic aneurysm, or dissection of an arterial aneurysm.

*Non-cardiovascular death:* Any death not covered by the above definitions, such as infection, malignant tumors, sepsis, respiratory causes, accidents, suicides, or deaths caused by trauma.

*Myocardial Infarction (MI):* Defined as ischemic symptoms or signs lasting for more than 30 minutes, with at least two consecutive leads showing new ST-T changes or Q waves or the appearance of a new left bundle branch block and elevated cardiac biomarker levels. Relevant significant changes in creatine kinase-MB (CK-MB) levels included CK-MB increasing again on the basis of peak decline of ≥ 25%, CK-MB increasing by more than 50% from the previous level, CK-MB increased by more than 2 times the normal upper limit in the absence of coronary intervention, and CK-MB exceeded 5 times the normal upper limit after PCI. Target vessel myocardial infarction refers to myocardial infarction events caused by the target vessel lesion.

Ischemia-driven target vessel revascularization: Nonplanned revascularization (PCI or coronary artery bypass grafting) of the target vessel treatment segment (DCB treatment area proximal and distal 5 mm) and upstream and downstream branches.

*Other Events:* Additionally, events, including ischemic stroke, bleeding events, hospitalization due to unstable or progressive angina, etc., will also be collected. Hospitalization due to unstable or progressive angina was defined as a final diagnosis of myocardial ischemia and the presence of the following conditions: ischemic discomfort or equivalent symptoms lasting at least 10 minutes at rest or equivalent symptoms requiring hospitalization within 48 hours of symptoms onset, or accelerated ischemic discomfort or equivalent symptoms after hospitalization within 48 hours. Additionally, at least one of the following criteria should be met: dynamic ST-segment depression, ischemia during stress testing, or significant epicardial coronary artery stenosis.

**Follow-up**

Participants in this study were scheduled for a total of four follow-up visits, occurring at the first, third, and ninth months and at the first-year post-inclusion. During these visits, comprehensive data collection will include a complete lipid profile, liver and kidney function tests, and information on any adverse cardiovascular events. Additionally, a telephone follow-up focused on adverse cardiovascular events will be conducted at the six-month mark. At the end of the first year, all patients underwent coronary angiography for reassessment, and OCT was performed to evaluate plaque morphology. The analysis factors included the fibrous thickness of the target coronary lesion, the lipid core arc of the target lesion, the minimum lumen area of the target lesion, and the proportion of vulnerable plaques in the target vessel.

Adherence to the follow-up schedule was mandatory for all patients. If rescheduling is necessary, subsequent follow-ups should align with the original timeline. Recognizing that certain circumstances may preclude scheduling follow-ups at the exact specified intervals, a flexibility window is allowed: ±7 days for the first month, ±14 days for the third month, ±30 days for the sixth month, ±30 days for the ninth month, and ±30 days for the first year.

**Safety assessment**

In this study, we recognize that the absence of scaffolding from DCB treatment presents risks, particularly the potential for significant elastic recoil of the vessel wall, which may result in acute vessel occlusion and thrombogenesis. Therefore, it is imperative to focus on critical safety endpoints, particularly regarding bail-out stenting.

We consider intermediate and non-flow-limiting vulnerable plaques to be relatively straightforward for interventional therapy, typically resulting in fewer adverse events and improved clinical outcomes compared to more complex lesions. Procedural techniques involving DCB must adhere to the guidelines set forth by the Asia-Pacific Consensus Group and the Third Report of the International DCB Consensus Group.

Pre-dilatation before DCB angioplasty should be executed using a non-compliant, cutting, or scoring balloon at a 1:1 balloon-to-vessel size ratio. Following lesion preparation, a 5- to 10-minute observation period is recommended, culminating in an angiogram to assess the adequacy of lesion preparation based on the following criteria: 1) ≤30% residual stenosis (visual assessment); 2) Thrombolysis In Myocardial Infarction (TIMI) flow grade of 3; and 3) the absence of flow-limiting dissection (NHLBI types D, E, and F).

Once these criteria are met, the DCB—extending at least 2-3 mm beyond the lesion on each side—should be inflated at nominal pressure for 60 seconds. An additional 10-minute observation period should follow DCB balloon withdrawal, leading to a subsequent angiogram to confirm satisfactory results. In instances where TIMI flow is less than 3 or severe dissections (types D, E, and F) occur, the implantation of a rescue drug-eluting stent will be mandated for bail-out treatment.

After the DCB procedure and prior to discharge, close monitoring of patient symptoms and electrocardiogram changes is essential for timely detection of any condition alterations. Repeat angiography will be recommended in cases of ischemia-driven dynamic ST-T changes, with stent implantation performed as needed.

The incidence of "rescue stent" implantation will be closely monitored and specifically analyzed statistically, under the oversight of an independent Data and Safety Monitoring Board.

**Data Management**

The data collection and management in this study were facilitated through an internet-based clinical research electronic management platform utilizing an electronic data capture (EDC) system specifically designed for clinical trials. This system is supplemented by case records (CRFs) and source documents integral to the trial processes. The data encompass a wide range of information, starting from baseline subject details such as demographic data and laboratory test results at enrollment. Additionally, parameters pertinent to trial procedures and lesion characteristics, covering aspects such as intervention pathways, characteristics of the culprit and target vessels, lesion length, degree of target vessel narrowing, reference target vessel diameter, and minimum lumen area both before and after intervention, will be also included. Information on the treatment of nontarget lesions and endpoint events will be also obtained.

Source documents serve as a crucial foundation, providing evidence of the patient's participation and verifying the integrity of the collected data. Researchers are responsible for archiving and storing these documents securely. It is imperative that the data transcribed from source documents to CRFs and the EDC system remain consistent. Any discrepancies identified must be thoroughly investigated and duly explained.

A comprehensive data security monitoring plan was established to meticulously record all adverse events in detail, ensuring proper handling and tracking until resolution or stabilization of the patient's condition. Serious adverse events, as well as unexpected incidents, are reported in a timely manner to the ethical review committee and relevant regulatory authorities in accordance with established regulations. The principal investigator undertakes regular cumulative reviews of all adverse events and has established an independent Data Safety Monitoring Committee to oversee the accumulated data on safety and efficacy endpoints. Additionally, if deemed necessary, meetings with research investigators are convened to evaluate the risks and benefits associated with the study. These meetings aimed to make informed recommendations on the continuation of the study, prioritizing the safety and rights of all participants.

**Handling of missing data**

The handling of missing data will follow the principles specified in the ICH-E9, and Guideline on Missing Data in confirmatory trials Guidelines.

Every effort will be made to collect all data. However, despite best efforts, missing or incomplete data may be reported. All missing or partial data will be presented in patient data listings, as they are recorded on the eCRF. Unless otherwise specified, as noted below, in general, no imputation of values for missing data will be performed.

1. Baseline covariates

Imputation methods based on variable distributions will be used to impute missing values for variables used in covariate analysis. Missing baseline covariates will be imputed using simple imputation methods in the covariate adjusted analysis based on the covariate distributions. For a continuous variable, missing values will be imputed from random values from a normal distribution with mean and SD calculated from the available sample. For a categorical variable, missing values will be imputed from random values from a uniform distribution with probabilities P1, P2, …, and Pk from the sample. Seed for the imputation is set as 128.

1. Efficacy outcomes

Missing data will not be imputed.

1. Safety outcomes

Missing data will not be imputed.

**Supplementary Table 1. Participating centers and the principal investigators at each center.**

| Participating centers | Principal investigators | Cities in China |
| --- | --- | --- |
| West China Hospital of Sichuan University | Yong He, Zhongxiu Chen | Chengdu, Sichuan |
| Zunyi First People's Hospita | Ranzun Zhao | Shanghai |
| Beijing Anzhen Hospital affiliated with Capital Medical University | Yong Zeng | Chaoyang, Beijing |
| Sichuan Provincial People ‘s Hospital | Gang Li | Chengdu, Sichuan |
| Chengdu Third People's Hospital | Lin Cai | Chengdu, Sichuan |
| Yan‘an Hospital of Kunming City | Qiang Xue | Kunming, Yunnan |
